# Development and Testing of a Two-UAV Communication Relay System

**DOI:** 10.3390/s16101696

**Published:** 2016-10-13

**Authors:** Boyang Li, Yifan Jiang, Jingxuan Sun, Lingfeng Cai, Chih-Yung Wen

**Affiliations:** Department of Mechanical Engineering, The Hong Kong Polytechnic University, Hong Kong, China; boyang.li@connect.polyu.hk (B.L.); jiang.uhrmacher@connect.polyu.hk (Y.J.); jingxuan.j.sun@connect.polyu.hk (J.S.); 11811766d@connect.polyu.hk (L.C.)

**Keywords:** two-UAV system, communication relay, mission control software, Ground Control Station

## Abstract

In the development of beyond-line-of-sight (BLOS) Unmanned Aerial Vehicle (UAV) systems, communication between the UAVs and the ground control station (GCS) is of critical importance. The commonly used economical wireless modules are restricted by the short communication range and are easily blocked by obstacles. The use of a communication relay system provides a practical way to solve these problems, improving the performance of UAV communication in BLOS and cross-obstacle operations. In this study, a communication relay system, in which a quadrotor was used to relay radio communication for another quadrotor was developed and tested. First, the UAVs used as the airborne platform were constructed, and the hardware for the communication relay system was selected and built up. Second, a set of software programs and protocol for autonomous mission control, communication relay control, and ground control were developed. Finally, the system was fully integrated into the airborne platform and tested both indoor and in-flight. The Received Signal Strength Indication (RSSI) and noise value in two typical application scenarios were recorded. The test results demonstrated the ability of this system to extend the communication range and build communication over obstacles. This system also shows the feasibility to coordinate multiple UAVs’ communication with the same relay structure.

## 1. Introduction

In recent years, as a result of decreased weights and sizes, reduced costs, and increased functionalities of different sensors and components, Unmanned Aerial Vehicles (UAVs) are becoming increasingly popular in a wide range of domestic applications (photography, surveillance, environment monitoring, search-and-rescue, etc.) [1,2,3]. As the market develops and the variety of applications expands, more requirements are now being imposed on extending the flight range and increasing the adaptability to the complex missions of UAV systems. In missions such are surveillance and search-and-rescue, UAV systems are usually required to operate in complicated environments while still being able to maintain uninterrupted communication with the ground control station for the purpose of reporting, monitoring, and control. For example, in the mountainous or high-density urban regions, the communication between UAVs and the ground control station can be easily interrupted, causing the ground control station to lose real-time data feedback from the UAVs, leading to mission failure. In addition, if the mission requires long flight range, the communication might also be interrupted by the distance.

Commercially available yet economical UAV communication solutions, however, have a number of limitations that potentially hinder their widespread application. First, the communication range of major civil UAV communication solutions is comparatively short. Telemetry modules, such as 3DR Sik2, XBee, and other Wi-Fi modules, which are common options for civil UAVs, have communication ranges that are limited to a few kilometers. Second, it is difficult to establish a stable data link when obstacles such as trees, tall buildings, or mountains separate the UAVs and GSC.

**Figure 1:**
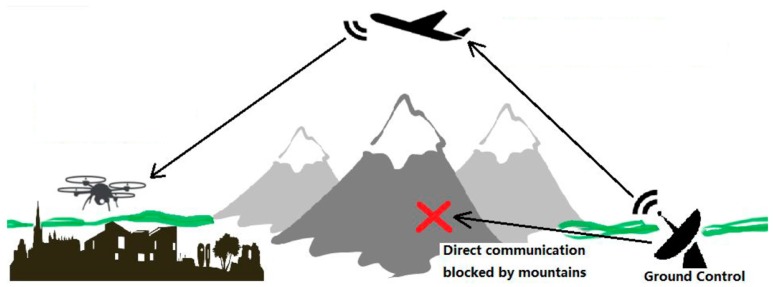
Application scenarios of UAV communication relay system.

In situations discussed above, it can be difficult to complete the mission with only one UAV deployed, while keeping cost and system complexity requirements met [4,5,6,7]. To harness the advantages of UAVs in these situations and minimize the drawbacks at the same time, an alternative solution is to deploy a multi-UAV system, which could utilize the inter-connectivity among multiple UAVs to maintain uninterrupted communication between every UAV and the ground control station. In this study, a UAV communication relay solution was developed, which uses relay and routing to extend the communication range and bypass obstacles at low cost. The objective was to develop and test a proof-of-concept two-UAV system that demonstrated the ability to relay radio communication. This system only consisted of two UAVs, but its design could accommodate the addition of more UAVs. The system uses one UAV as communication relay point and enables the other UAV to operate in areas where direct communication with ground control cannot be established. This configuration allows communication to be established across obstacles or over a distance exceeding the range of the onboard radio transceiver, both of which will be demonstrated in field tests later. The typical application scenario of this system is shown in Figure 1 schematically.

The paper is organized as follows. Section 2 introduced the related works. Section 3 introduces the hardware configurations of the multiple UAVs communication relay system. Section 4 discusses the software aspects of the system. In Section 5, both the indoor and outdoor tests methods and results are presented. A conclusion of the work is given in Section 6.

## 2. Related Works

Numerous researches have been performed in UAV communication relay scheme. In the conceptual study of UAV based military communication relays, Pinkney et al. [8] described a multi-year airborne communication range extension program plan which was primarily used to support the Battlefield Information Transmission System. The main objective of the program was to provide the beyond line of sight (BLOS) communications capabilities without the help of scarce satellite resources. Their range of coverage can vary from 50 to 100 miles for UAVs of interest. Rangel et al. [9] developed a multipurpose aerial platform using a UAV for tactical surveillance of maritime vessels. They concluded that the communication relay using a UAV system is a low-cost application which could provide air dominance with satisfied communication range with lost cost resources. In Orfanus et al. [10], the use of the self-organizing paradigm to design efficient UAV relay networks to support military operations was proposed.

Abundant previous studies have focused on the algorithms for vehicle position assignment and network configuration. Choi et al. [11] developed guidance laws to optimize the position of a Micro-UAV which relayed communication and video signals when the other UAV is out of radio contact range with the base. Zhan et al. [12] investigated a communication system in which UAVs were used as relays between ground terminals and a network base station. Their work mainly focused on developing the algorithm for optimizing the performance of the relay system. In [13,14], Cetin and coworkers proposed a novel dynamic approach to the relay chain concept to maintain communication between vehicles in the long-range communication relay infrastructure. Their path planning method was based on the artificial potential field.

Many other researchers used simulation methods to propose and test the relay systems. Hauert et al. [15] conducted 2D simulations of the design on a swarm system which does not make use of positioning information or stigmergy for application in aerial communication relay. Jiang and Swindlehurst [16] simulated the dynamic UAV relay positioning for multiple mobile ground nodes and multi-antenna UAVs. In [17], a relay network composed of UAVs keeping the Wireless Sensor Network connected was proposed. They also used cooperative multiple input multiple output (MIMO) techniques to support communication. Ben Moussa et al. [18] provided a simulation-based comparison between two strategies for an autonomous mobile aerial node communication relay between two mobile BLOS ground nodes. Lee et al. [19] proposed an airborne relay-based regional positioning system to address pseudolite system limitations. They conducted simulations and demonstrated that the proposed system guarantees a higher accuracy than an airborne-based pseudolite system.

For experimental studies, most of the previous works were based on IEEE 802.11b (Wi-Fi) standard. Brown et al. [20,21] implemented a wireless mobile ad hoc network with radio nodes mounted at fixed sites, ground vehicles, and UAVs. One of their scenarios was to extend small UAVs’ operational scope and range. They measured detailed data on network throughput, delay, range, and connectivity under different operating regimes and showed that the UAV has longer range and better connectivity with the ground nodes. Jimenez-Pacheco et al. [22] also built a mobile ad hoc network (MANET) of light UAVs. The system has implemented a line of sight dynamic routing in the network. Morgenthaler et al. [23] described the implementation and characterization of a mobile ad hoc network for light flying robots. Their UAVNet prototype was able to autonomously interconnect two end systems by the airborne relay. Asadpour et al. [24,25] conducted simulation and experimental study of image relay transmission for UAVs. Yanmaz et al. [26] proposed a simple antenna extension to 802.11 devices for aerial UAV nodes. They tested the performance of their system and showed that 12 Mbps communication can be achieved at around 300 m. Yanmaz et al. [27] also did a follow up work on a two-UAV network using 802.11a. In this work, they showed that high throughout can be achieved using two-hop communications. Johansen et al. [28] described the field experiment with a fixed wing UAV acted as a wireless relay for Underwater Vehicle. They only tested the download data rates. Notably, although the Wi-Fi network is convenient to build multiple access points, its communication range is highly restricted and a large number of nodes are needed for long range applications. One of the advantages of a Wi-Fi network is its high throughput. However, for UAV telemetry communication, the distance is a more important requirement than the transmission speed.

For experimental works without the use of Wi-Fi network, Guo et al. [29] analyzed the use of small UAVs for 3G cellular network relay. They tested the upload and download throughputs and the ping time in both urban and rural environments. Deng et al. [30] developed the UAVs and the communication relay system for power line inspection. Their system consisted of multi-platform UAVs and multi-model communication system for image/video transmission. However, they did not test the communication range or quality of their system. The only one work used 433/915 MHz as the communication frequency was Willink et al. [31] who measured the low-altitude air-to-ground channel at 915 MHz. They mainly focused on studying the spatial diversity to and from the airborne node and also measured time delay and received power.

For all above-mentioned works, The UAVs in their systems did not exchange messages with the GCS by the relayed communication, which means the UAVs were used as carriers to realize communication relay for other systems rather than acting as the active parts in the relay systems. Therefore, the applications would be constrained if UAVs lost connection with the GCS. The highlights of our work are the building of an interface and protocol with the flight controller and direct monitoring and control of the UAVs with relayed communication. This study provides a viable and economic way, with detailed hardware and software methods described, to construct a prototype of the UAV communication relay system.

## 3. Hardware Platform

**Figure 2:**
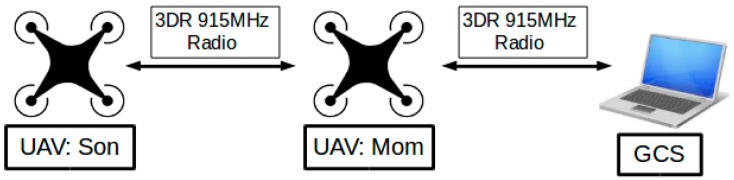
Whole architecture of the communication relay system.

In this section, the hardware construction of the miniature two-UAV system is introduced. A miniature UAV relay system with the ability to relay radio communications was built. The hardware mainly consisted of three communication nodes: a GCS; a UAV that relays radio communication, which was named “Mom”; and a second UAV named “Son”, whose communication with the GCS was relayed through the UAV “Mom”. The schematic of the major hardware platform for the “Mom and Son” relay system is illustrated in Figure 2 and the detailed communication subsystem will be introduced in Section 3.3 later.

### 3.1. System Architecture

**Figure 3:**
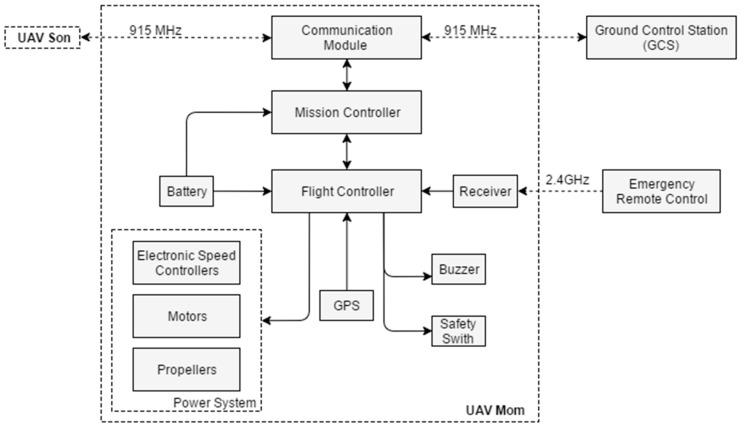
System hardware architecture.

The relay system can be conceptualized as the following major functional blocks, with the corresponding hardware architecture shown in Figure 3. The figure mainly describes the main components in UAV “Mom” while all other UAVs have the same hardware architecture.

- **Aircraft**: This is the body of the UAV, usually consisting of structural components, power system, and battery. In the test system, the aircraft were constructed using commercially available frame kits. Both of the UAVs, i.e., “Mom” and “Son”, were selected as quadrotors for easily launch and recovery ability during the development period. Communication relay systems, however, are not limited to quadrotors and can be integrated into any desired airborne platform such as fixed wing UAV for long endurance and distance relay service.
- **Flight Controller**: This includes main flight control boards as well as the necessary sensors, that together provide attitude and position control for the UAVs. In this test system, 3DR Pixhawk kit [32] was selected as the main flight controller. The controller has an internal IMU, barometer, external GPS, and a compass for attitude and position measure. It also includes safety switch, buzzer, LEDs, and different kinds of interfaces combined to provide flight control.
- **Communication Module**: This functional block directly handles wireless radio communications, and consists of radio transceivers and antennas. The paired modules will connect automatically and transmit data instructed by the mission controller.
- **Mission Controller**: This functional block is a microcomputer running Linux operation system and mainly responsible for the following functions: (1) conducting communication flow control, message routing, and message error checking; (2) monitoring UAV flight conditions and reporting to GCS; and (3) translating mission commands from GCS to low-level instructions, which are directly supplied to the flight controller.
- **Ground Control Station**: This functional block refers to a Windows-based laptop running the GCS software to send commands to all the UAV nodes and monitor the status of all UAVs.
- **Emergency Remote Controller**: The remote controller is connected to the flight controller via 2.4 GHz wireless communication. In normal operation, all the commands and status can be set by the GSC point. This device has switch to directly takeover the control of UAVs in case of emergency situation in the debugging process.

### 3.2. Communication Subsystem

To construct the communication subsystem, a number of commonly used wireless communication technologies were considered as the possible candidate: IEEE 802.11n Wi-Fi, 3G/4G-LTE mobile network, and 433 MHz/915 MHz radio. Wi-Fi can provide a reliable, high-speed connection, but has a comparatively small range. Typical Wi-Fi routers have an effective range of fewer than 100 m, rendering them unsuitable for long-range outdoor missions. The applicability of the 3G/4G-LTE mobile network relies heavily on the strength of mobile phone signal, which varies greatly in different locations, and is not always available in remote areas where surveillance and rescue missions are likely to take place. A 433 MHz/915 MHz radio can be operated without a license in many parts of the world, and can be implemented with easily accessible, low-cost transceivers (for example, 3DR Radio Telemetry as discussed below). A range of a few kilometers could be achieved. The radio can also be deployed at any desirable site without the need for supporting infrastructure. These features give 433 MHz/915 MHz radio advantages in various outdoor missions. The frequency options, 433 MHz or 915 MHz, can be selected to ensure compliance with local radio regulations.

Based on the discussion above, to establish communication between the UAVs and GCS and to facilitate the communication relay system, 3DR 915 MHz Radio Telemetry (3DR Radio) [33] was selected as our radio transceiver hardware. 3DR Radio, the built-in processor of which runs open-source SiK firmware [34], has the following useful features that enable communication relay:

- Built-in time division multiplexing (TDM) support.
- Supports frequency hopping spread spectrum (FHSS) among a configurable number of channels (configured to 50 channels in this study). Its frequency hopping sequence is user-configurable.

**Figure 4:**
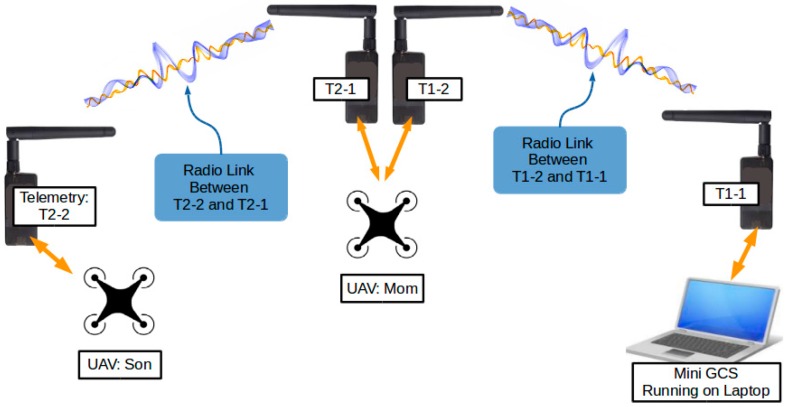
Radio communication relay setup.

Figure 4 illustrates the radio communication relay setup. Four 3DR Radios were used, numbered T1-1, T1-2, T2-1, and T2-2. Table 1 gives the details of the configuration and installation of the telemetries. T1-1 and T1-2 carried communication between GCS and UAV “Mom” exclusively, whereas T2-1 and T2-2 carried communication between UAV “Mom” and UAV “Son”.

**Figure 5:**
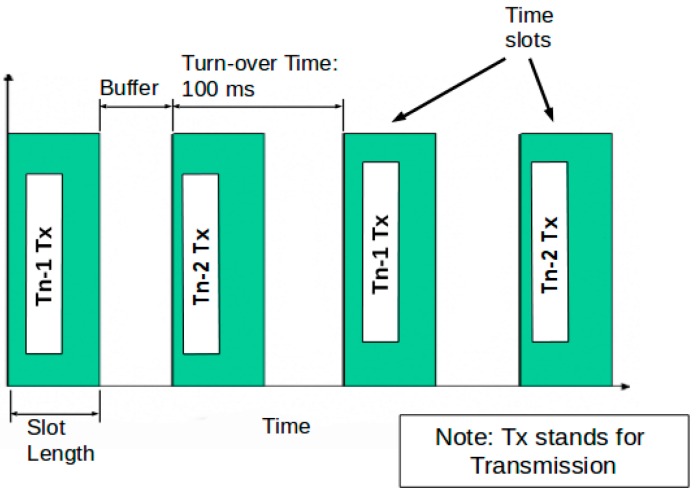
Time division multiplexing (TDM) working principle.

**Figure 6:**
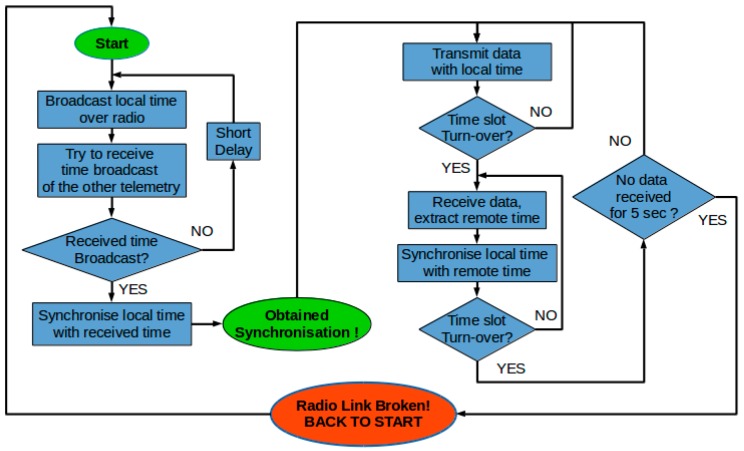
TDM process flow chart.

A combination of TDM and FHSS techniques was used to ensure that the presence of multiple radio telemetries did not lead to radio-frequency interference. By default, telemetries Tn-1 and Tn-2 (where *n* = 1, 2…) formed one radio link between each another. On power-up, Tn-1 and Tn-2 first searched for synchronization signals from each another; these signals were used to synchronize the internal clocks of both telemetries. Once the clocks were synchronized, the telemetries then started a TDM process, in which Tn-1 and Tn-2 alternately transmitted data over radio, and listened to incoming data when not transmitting. The alternation of a transmission window occurred at a fixed frequency, and the time between consecutive alternations was referred to as the turnover time. The selection of the turnover time depends on the time required to transmit messages. On one hand, the turnover time should be long enough to allow an adequate number of messages to be transmitted in a single transmission window. On the other hand, the turnover time should not be too long, otherwise, the other telemetry would have to wait longer before it can transmit its own messages, causing delays in information update, and compromising the synchronization accuracy between telemetries. The value of the turnover time could be tuned based on the above-mentioned principles. In this research, the turnover time was set to 100 ms, which ensured stable operation of the communication relay function. Values ranging from 90 ms to 140 ms have been tested and could provide similar results. This process is illustrated in Figure 5 and Figure 6.

**Figure 7:**
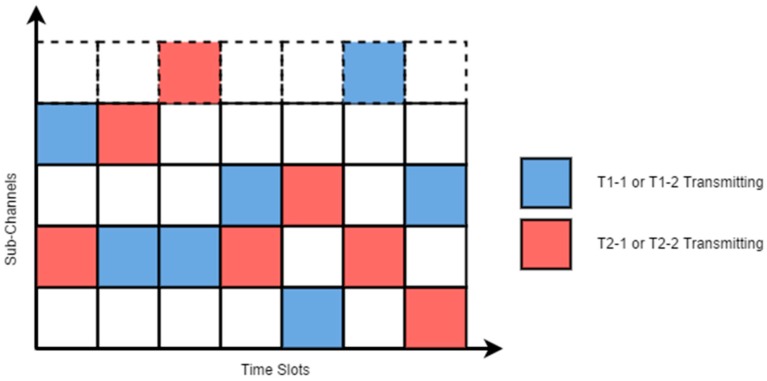
Frequency hopping of telemetries.

In addition to the TDM process, the transmission of telemetries Tn-1 and Tn-2 were frequency-hopped. The telemetries transmitted in one of the 50 sub-channels that were obtained by dividing the 915 MHz band into 50 equal portions. On the occurrence of the alternation of a transmission window, both Tn-1 and Tn-2 simultaneously hopped to a new sub-channel, which was determined by a random channel hopping sequence shared between Tn-1 and Tn-2. As shown in Table 1, T1-1 and T1-2 were configured to use a different hopping sequence than T2-1 and T2-2. Therefore, although multiple devices might be transmitting radio waves at the same time, the sub-channels in which they are transmitting are different, as illustrated in Figure 7.

### 3.3. Mission Control Subsystem

The control of key onboard functions: communication relay, flight status monitoring, and mission control, is centrally managed by the mission control subsystem, which UAV “Mom” and “Son” both carry onboard. The onboard mission control subsystem is mainly in charge of the following functions:

- Executing radio communication frame processing and relay control.
- Decoding commands contained in frames, and translating the commands into low-level instructions to the UAV’s flight controller.
- Monitoring UAV flight status, and reporting to the GCS on a regular basis.
- Handling ad hoc functions that specific missions necessitate, for example, image processing, dynamics flight route planning, smart target identification, etc.

**Figure 8:**
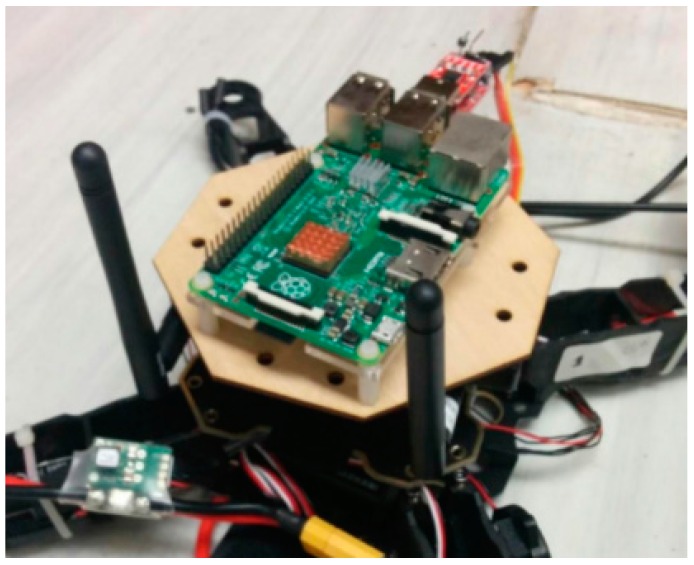
Onboard computer module.

The above-mentioned functions require the UAVs to be equipped with an on-board computer with adequate processing power. In this research, Raspberry Pi 2 Module B (Rpi 2B) was chosen as the onboard computer for both UAVs because of its relatively small size, strong processing power, and abundant IO port. Figure 8 shows the on-board computer module, with Rpi 2B mounted on top.

**Figure 9:**
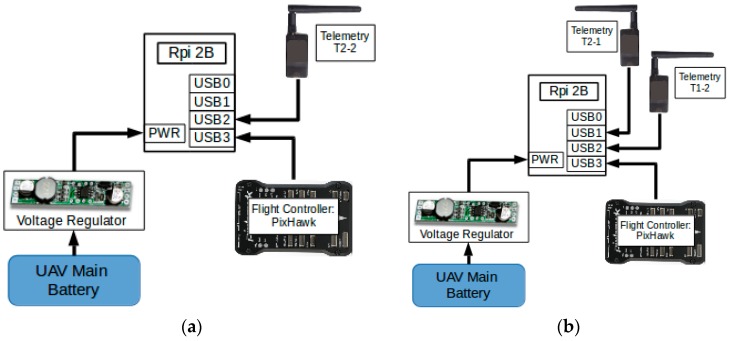
Hardware connection diagram of (**a**) UAV “Son” and (**b**) UAV “Mom”.

The onboard computer needs to frequently exchange data with and acquire information from other onboard devices: radio telemetries, the flight controller, the GPS module, and various onboard sensors. The onboard computer communicates with the peripheral devices through USB ports. Rpi 2B readily supports USB protocol and has four USB ports available. Most peripheral devices, however, utilize a low-level communication method named Universal Asynchronous Receiver and Transmitter (UART). Therefore, a protocol conversion module based on CP2102 microchip was wired between the USB ports of Rpi 2B and the peripheral devices, which handles the conversion between the USB protocol and the UART protocol. Figure 9 shows the hardware connection diagrams of the onboard computer module with other subsystems. The Rpi 2B of “Son” was connected to two USB devices which are T2-2 and the flight controller. The Rpi 2B of “Mom” was connected to three USB devices including T1-2, T2-1, and the flight controller.

The onboard computer module is powered by the UAV’s main battery and is switched on when the UAV is powered up. After power-up, the onboard computer module then automatically executes a self-initialization program, which configures necessary system parameters and starts up a set of software programs. The programs then run on the onboard computer module and execute functions such as communication relay control and mission control. Details about software structure and realization of the communication relay subsystem will be discussed in Section 4.

## 4. Software Development

The software developed in this project includes mission control software running on the mission control board and Mini GCS running on the GCS laptop. The mission control software is in charge of analyzing the target node as well as the command contents of each message it receives. If the ID of the messages represents other UAV nodes, the software will bypass this message to the corresponding targets. If the ID of the message corresponds to the receiving UAV itself, the software will further resolve the command data in the message and send it to the flight controller. The Mini GCS was developed to better coordinate the multiple UAV system. The condition of each UAV will be displayed on the screen including the flight mode, the GPS position, the attitude etc. The Mini GCS was also used to send commands to each UAV node by the user friendly Graphic User Interface (GUI).

### 4.1. Data Framing for Communication Relay

**Figure 10:**
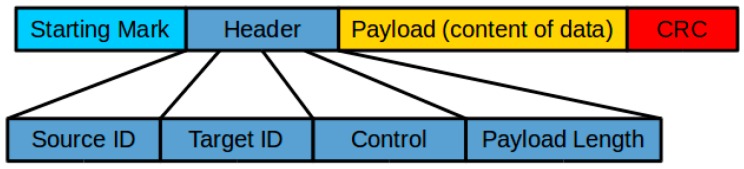
Format of frame.

To facilitate the communication relay, the messages and data to be exchanged between the UAVs and GCS were grouped into frames, the format of which is shown in Figure 10. Each communication node (“Mom”, “Son”, and GCS) was given a unique ID. The header of the frame contained two fields, “Source ID” and “Target ID”. “Source ID” indicated the node from which the frame was transmitted, and “Target ID” indicated the target receiver of this frame. This mechanism enabled the identification of the sender and receiver of a data frame, which was important to the realization of frame routing.

### 4.2. Mission Control Software

**Figure 11:**
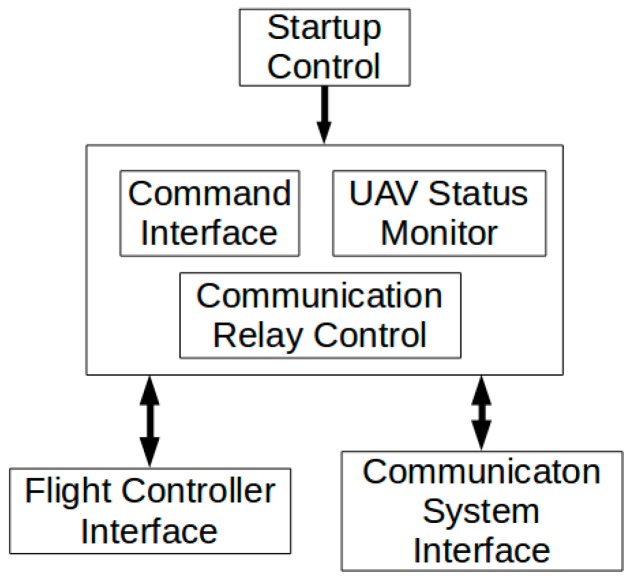
Block diagram of mission control software.

The mission control software, which runs on the UAV’s on-board computer, consists of the following seven modules, and the software’s block diagram is shown in Figure 11.

1. Flight Controller Interface: manages direct communication with Pixhawk flight controller over USB port.
2. UAV Status Monitor: monitors flight and mission parameters of the UAV, and reports them regularly to the GCS.
3. Command Interface: translates user commands into low-level instructions to the flight controller; records and reports command execution status.
4. Communication System Interface: manages low-level message traffic between mission control subsystem and ration telemetries; executes message framing, frame reception, and error checking.
5. Communication Relay Control: controls the relay of radio communication.
6. Startup Control: bootstraps the whole mission control software system during UAV power-up process.

### 4.3. Software Realization of Relay Process

The presence of a relay implies that the relaying node (UAV “Mom” in this case) needs to handle a frame routing. When processing received frames, the UAVs made use of the “Source ID” and “Target ID” fields to filter and/or route the frames in the following manner.

- UAV “Son” is the terminal receiver, which only receives frames over the relay from “Mom.” Therefore, “Son” only saves for further processing frames whose “Target ID” is equal to the ID of “Son” itself.
- UAV “Mom” functions as a communication relay node, but it also receives control commands from GCS. Therefore, “Mom” will save for further processing frames whose “Target ID” is equal to the ID of “Mom” itself, and will re-transmit frames targeted at “Son” over Radio T2-1.

**Figure 12:**
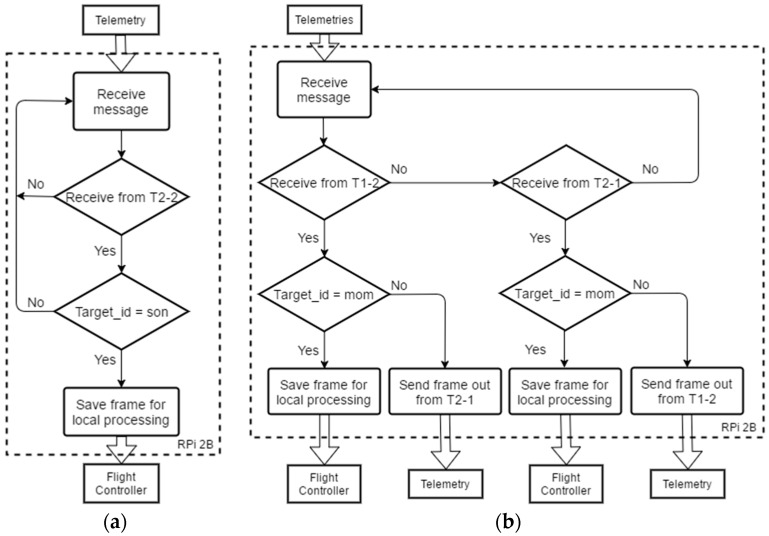
Flow chart of mission control software for (**a**) UAV “Son” and (**b**) UAV “Mom”.

The above-mentioned frame routing logic is elaborated in the following pseudocode (Algorithms 1 and 2) and flow chart in Figure 12.

**Algorithm 1:** Frame Processing Logic, UAV Son*For (true) do:*  *if (frame receive from T2-2) do:*       *if (frame_target_id == id_son) do:*         *save frame for local processing.*       *else do:*        *discard frame.*       *end*  *end**end*

**Algorithm 2:** Frame Processing Logic, UAV Mom*For (true) do:*  *if (frame receive from T1-2) do:*       *if (frame_target_id == id_mom) do:*         *save frame for local processing.*       *else if (frame_target_id == id_son) do:*         *send frame out from T2-1.*       *end*  *else if (frame received from T2-1) do:*       *if (frame_target_id == id_mom) do:*         *save frame for local processing.*       *else if (frame_target_id == id_GCS) do:*         *send frame out from T1-1.*       *end*  *end**end*

The last field of the frame contained a four-byte cyclic redundancy checking (CRC) code, which was computed using the IEEE 802.3 32-bit CRC polynomial. This CRC code was important because it helps verify data frame integrity and protect the system from the influence of error-corrupted data or messages. Error-corrupted data or messages could be dangerous, as they might be mistakenly understood by the UAV’s mission control subsystem as wrong commands, resulting in unpredictable and potentially dangerous behavior.

### 4.4. Mini GCS

A ground control software, named “Mini GCS”, was developed in-house. Mini GCS is an integrated UAV control interface that is compatible with Windows 7, 8, and 10 systems. The functions of Mini GCS include handling data exchanges with UAVs, displaying information about the UAVs, and providing an interface for users to send commands to the UAVs. It allows users to monitor the status of both UAVs, and to control the mission of each UAV from a single, integrated interface, which makes it a crucial communication node in the communication relay network. Mini GCS was implemented using C#, and the graphic user interface (GUI) was constructed using Microsoft Windows .NET framework. Because the main focus of this work is to demonstrate a signal relay system for the UAV applications, details of this Mini GCS software are not emphasized in the manuscript and the source code is included in the section of Supplementary Materials for reference.

**Figure 13:**
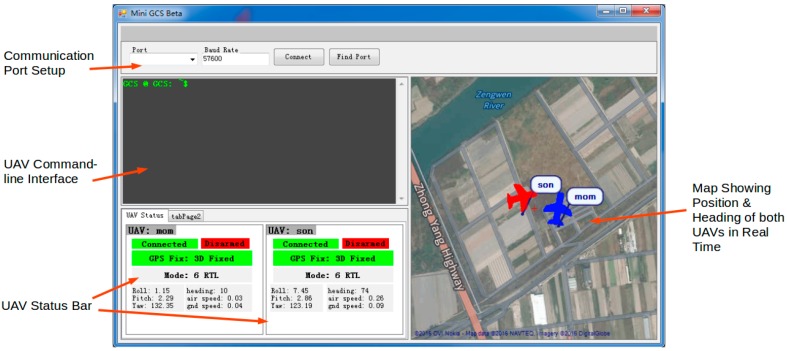
Graphic user interface (GUI) of Mini GCS.

Figure 13 shows a screenshot of Mini GCS. A number of features are available.

- **UAV Status Display:** displays to users a selected set of important information about each UAV, including communication status, GPS status, flight mode, altitude, speed etc.
- **UAV Command Line:** a command line interface that allows users to send specific commands to any UAV nodes in the network such as takeoff, land, return to launch, etc.
- **UAV Position Display:** displays the real-time position and heading of both UAVs in a map of the mission area.

## 5. Performance Tests

To verify the performance of the system, a series of indoor and outdoor tests were carried out. First, the data loss rate and bit error rate (BER) were measured in the laboratory environment to choose the suitable data transmission rate for the relay system. Then, for outdoor tests, Received Signal Strength Indicator (RSSI), a measurement of the power present in a received radio signal, was measured. The RSSI value scales as 1.9× the dBm signal strength, plus an offset, which means the RSSI is proportional to the dBm [35]. We tested both UAVs in two typical application scenerios in Hong Kong: one was to cross obstacles and the other was to extend the communication range. Finally, a BLOS demonstration was conducted to show the rundown and results of a whole application process in Taiwan. Notably, when the transmission power of the telemetry module is set to the maximum value, which is 20 dBm (100 mW), the typical range achieved using standard configuration and antenna is around 500 m. Due to the stringent flight regulation and the area constraint of the test site in Hong Kong, it is almost impossible for the authors to test the beyond-line-of-sight flight and the maximum power relay which would exceed the allowed flight area. Therefore, to demonstrate the relay function in Hong Kong, the authors have adjusted the transmission power of the radio to its minimum value (1/10 of the max.) so that the radios will disconnect at about 100 m. In this way, the relay methods can be tested in a relatively small area, otherwise, the telemetry modules will always be connected. In missions requiring long distance communication, the transmission power can be easily turned to a maximum value to get the best communication range.

### 5.1. Indoor Tests

To investigate the reliability of our radio communication relay, the data loss rate and the BER were first tested indoors. Verifying the data loss rate and BER in an indoor environment under different data rates gave us knowledge about the usable bandwidth of the system and helped us estimate the maximum data rate that can be used in the flight tests. The GCS was configured to transfer data at a fixed data rate, and the statistics of the received data at UAV “Son” were measured. Two aspects of the data transfer were studied: the data loss rate, which is the percentage of data that was lost during transmission (data that was transmitted from GCS but was not received by UAV “Son”); and the BER, which was the percentage of data that was received by “Son” but did not pass the CRC test.

**Figure 14:**
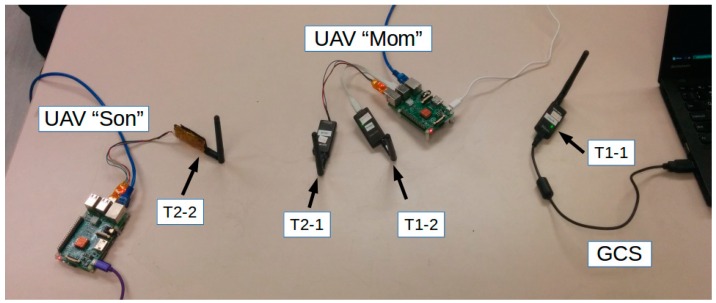
Radio communication relay testing setup.

**Figure 15:**
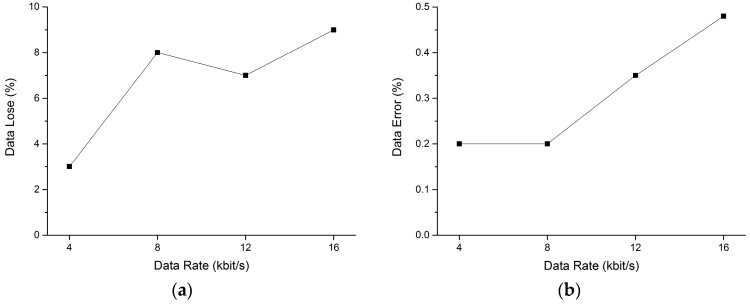
(**a**) Percentage of data loss and (**b**) bit error rate with respect to data rate.

Tests were conducted at four different data rates: 4 kilobits per second (kbps), 8 kbps, 12.8 kbps, and 16 kbps. Figure 14 shows the setup of the test. The percentages of data loss and BER with respect to the data rate are shown in Figure 15.

The tests indicated that when the data rate was equal to or higher than 4 kbps, the data lost percentage rose from 3% to 9% and the data error rate rose from 0.2% to 0.5%, approximately. A higher data rate resulted in both more data loss and a higher chance of data transmission errors. Andre et al. [7] estimated that the throughput of typical UAV mission commands is less than 10 kbps. In our tests, the current used data rate for a two-UAV system was about 8 kbps. Then, for communication relay applications with this system and configuration, a data rate of 8 kbps is recommended to ensure an acceptable compromised data lose and error rate.

### 5.2. Cross-Obstacle Test

**Figure 16:**
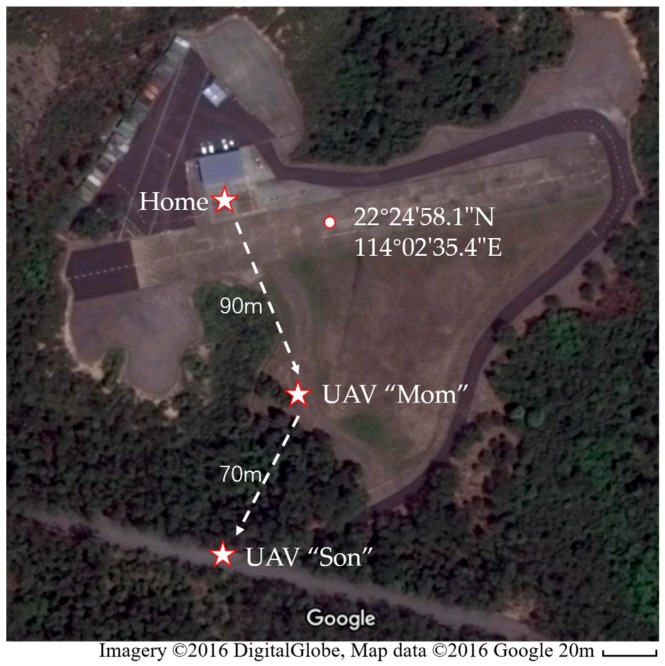
Final positions of nodes in the Google map in the cross-obstacle tests test in Hong Kong.

**Figure 17:**
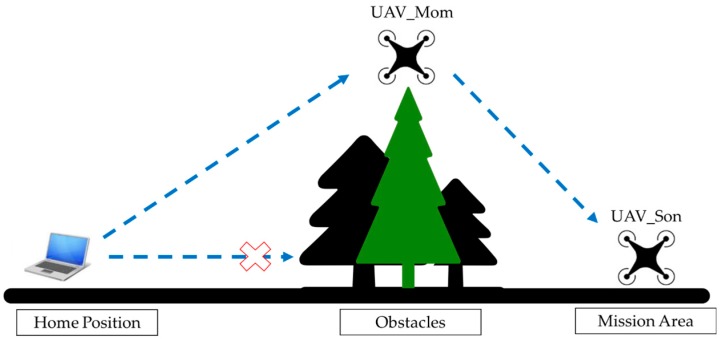
Schematic of cross-obstacle communication relay flight test.

The outdoor flight tests were first conducted in the International Model Aviation Center of the Hong Kong Model Engineering Club (22°24′58.1″ N 114°02′35.4″ E). Figure 16 shows the Google map of the test site and final positions of the UAVs in the test. In the simulated cross-obstacle communication relay test, the UAV “Son” was designed to conduct a mission that takes off from the “Home Position”, flies over a part of the forest with tall trees, and then lands on the road across the forest (UAV “Son” in Figure). The tall trees would block the communication signal from GCS to UAV “Son”. Then the UAV “Mom” took off and loitered at the UAV “Mom” position, which was above the forest. The UAV “Son” would recover the communication with home GSC with the help of relay point UAV “Mom”. The relative positions of all the communication nodes can be more clearly conceived in the schematic shown in Figure 17.

**Figure 18:**
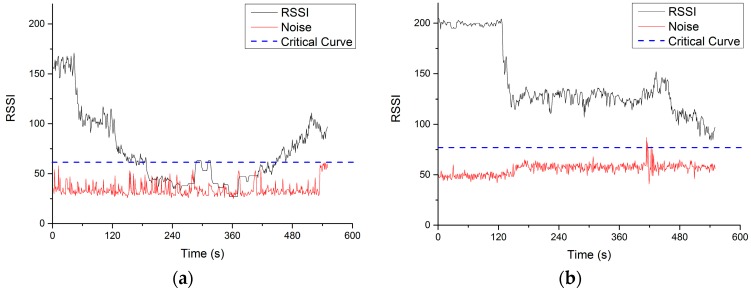
Received Signal Strength Indicator (RSSI), noise and critical curve of both (**a**) UAV “Son” and (**b**) UAV “Mom”.

RSSI and noise of both UAV “Son” and UAV “Mom” were recorded and shown in Figure 18. RSSI can reflect the strength of wireless signal received by the UAV “Mom” and UAV “Son”. After several tests, we found that only when RSSI value is 20 scale (about 7 dBm) higher than the average noise value can the wireless modules keep a stable connection. Therefore, a dashed blue line is added to each figure to indicator when the connection is lost or unstable. The test started at time 0 s, when UAV “Son” took off and flew to the mission area. The duration of the whole test mission was about 10 min. Since 120 s, the UAV “Son” reached the area behind the tall trees so the RSSI decreased significantly until it reached the noise level and the connection with the GCS was lost. At this moment, the UAV “Mom” took off and flew to the relay point. Before 120 s, UAV “Mom” stayed at home near the GSC so the RSSI remained at a high value. After take off, the RSSI of UAV “Mom” decreased but still remained much higher than the noise, which means that the UAV “Mom” kept connecting with the GSC. During the 180 to 420 s, the UAV “Son” flew over the forest and landed on the road. In the Figure 17a, the RSSI fell to the same scale with noise value. In this period, the UAV “Son” remained disconnected with GCS. After the time 420s, the UAV “Mom” flew close to UAV “Son” and relayed the communication to help UAV “Son” reconnect with the GCS. Then the RSSI value of “Son” recovered. The UAV “Mom” continually flied to UAV “Son” (away from the GCS) for a while which caused further increase of RSSI of “Son” and decrease of “Mon” at the end period.

### 5.3. Extend Distance Test

**Figure 19:**
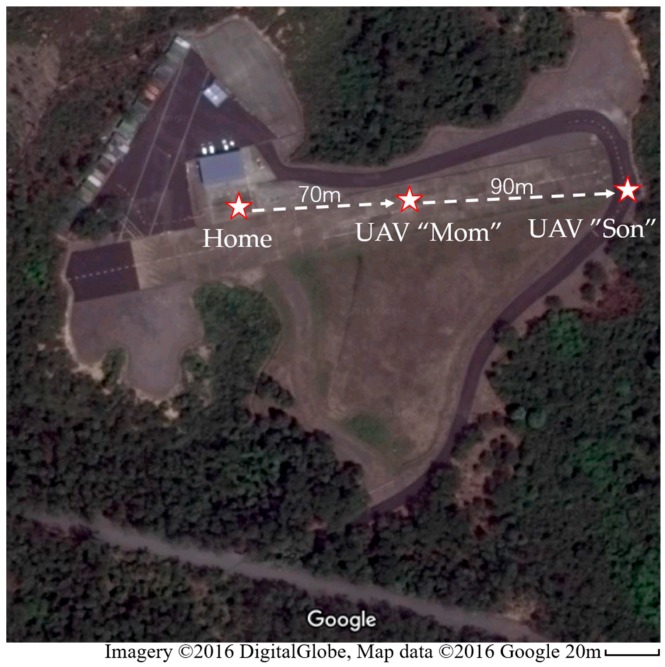
Final positions of nodes in the Google map in the extended distance test in Hong Kong.

In the communication distance extension tests in Hong Kong, the UAV “Son” was firstly given a mission to fly away as far as possible until the connection was lost. After recording the lost position, which is about 100 m, the UAV “Son” continued to fly away for about 60 m and stayed at the position, waiting for the UAV “Mom” to relay the communication. Thereafter, the UAV “Mom” took off and flew to the direction of UAV “Son” until the connection was rebuild. The final positions of UAV nodes in this test is shown in Figure 19. With the help of a relay node, a 160 m total connection was achieved.

**Figure 20:**
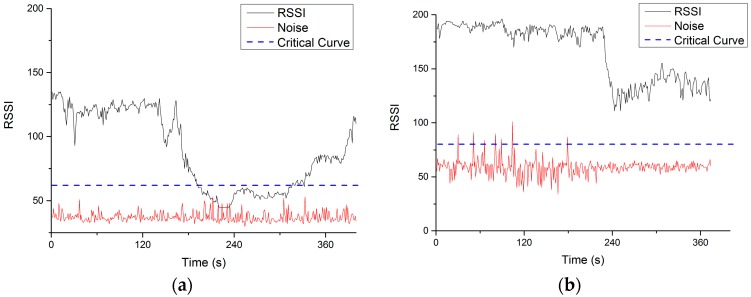
RSSI, noise, and critical curve of both (**a**) UAV “Son” and (**b**) UAV “Mom”.

The RSSI, noise and critical curves during the full test are shown in Figure 20. As is similar with the cross obstacle test, the communication between UAV “Son” and GCS was first lost at about 180 s and finally reconnected at about 300 s when the UAV “Mom” takeoff and flew near to UAV “Son”. The drop of RSSI of UAV “Mom” was due to its takeoff and flying away from home position.

### 5.4. Beyond-Line-of-Sight Tests

**Figure 21:**
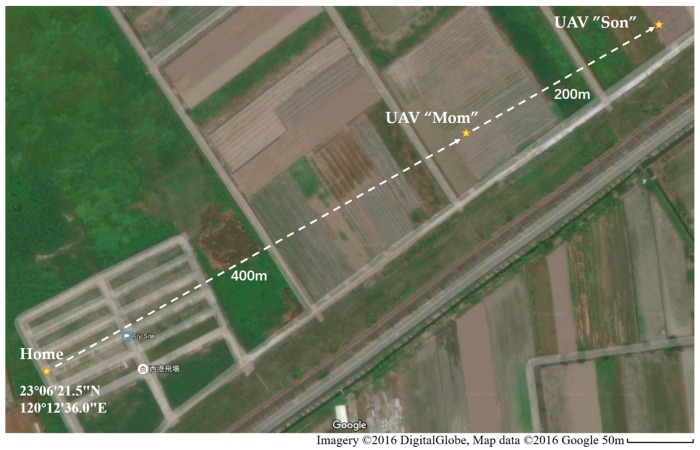
Final positions of nodes in the Google map in the extended distance test in Taiwan.

To further verify the reliability of the communication relay system, including mini GCS, in a beyond-line-of-sight missions, outdoor flight tests were conducted in Xigang flight site, Tainan City, Taiwan (23°6′21.5″ N, 120°12′36.0″ E). In this test, the transmission power of the telemetry modules were turned to the maximum value to get the longest communication range. The Google map of the test site and final positions of the UAVs in the test are shown in Figure 21.

The rundown of the typical flight mission is given below:

1. All UAVs (“Mom” and “Son” in this case) are powered up.
2. Start GCS software “Mini GCS”.
3. Wait for communication relay network to start. The startup process is automatic and normally takes less than three minutes.
4. Using “Mini GCS”, command “Son” to take off and start the execution of a preprogrammed mission, which brings “Son” to a destination far enough for the radio communication between “Son” and “Mini GCS” to break.
5. Wait until the communication between “Son” and “Mini GCS” breaks due to distance.
6. Using “Mini GCS”, command “Mom” to take off and move into the proximity of the destination area of “Son”. “Mom” automatically performs a communication relay when it moves toward “Son” and reconnects.

**Figure 22:**
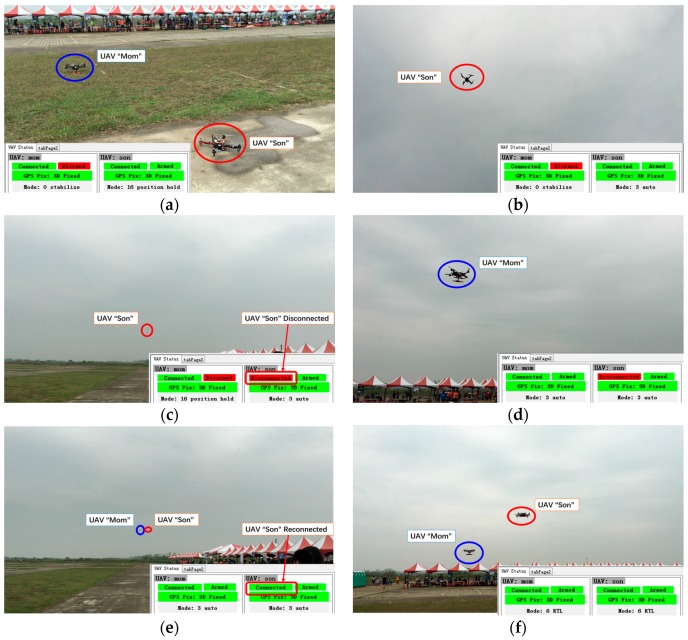
A rundown of flight test (**a**) Pre-takeoff setup of flight test; (**b**) UAV “Son” starts the mission; (**c**) “Son” is out of radio range and communication with it is lost; (**d**) UAV “Mom” is deployed to the relay position; (**e**) Communication with “Son” is re-established after being relayed through “Mom”; (**f**) “Mom” and “Son” return to launch position; the mission is completed.

The rundown of the test is shown as a series of pictures in Figure 22. A video clip demonstrating the complete test procedure is given in the Supplementary Material. In the video demonstration, UAV “Son” started a pre-defined mission. The mission is to fly away for about 600 m until the UAV was BLOS in Figure 22c. When the connection between “Son” and GCS was lost (indicated by the green “Connected” icon changing to the red “Disconnected”), “Mom” was deployed to a designated relaying position (about 400 m away from home position). Once “Mom” reached the relaying position, communication between “Son” and GCS was re-established automatically and was not lost again before the end of the mission.

Eight flight tests were conducted. In all of the tests, the system was able to re-establish communication between “Son” and “Mini GCS” through the relay of “Mom”. When “Mom” reached the designated relaying position, the communication connection between “Son” and “Mini GCS” was adequately stable for mission operations. The successful rate of the communication re-establishment is 100%. The tests demonstrated that: (1) the system is capable of maintaining uninterrupted communication between GCS and an end node once the relaying node is properly deployed; and (2) connectivity between GCS and the end node can be automatically re-established when the relaying node reaches the proper relaying position, even if it has been previously lost due to an inadequate relay.

## 6. Conclusions and Future Development

In this study, major attention was given to implementing a functional and reliable proof-of-concept UAV communication relay system that uses commercially available hardware components combined with customized software. In the prototype system, two quadrotors, each mounted with a Pixhawk flight controller and a Raspberry Pi 2 Model B microcomputer were used as the aerial platforms. The onboard relay control protocol and program were developed to realize UAV communication extension and cross-obstacle communication. The GSC software was also developed to facilitate the two-UAV relay operations. A series of indoor and outdoor tests were carried out. The indoor tests aimed to determine the suitable data transmission rate, while the outdoor tests demonstrated reliable communication performance in two proposed application scenarios in Hong Kong and one BLOS mission simulation in Taiwan. All the outdoor tests showed the system’s capability of maintaining good quality real-time communication through relays, and automatically recovering from communication losses.

**Figure 23:**
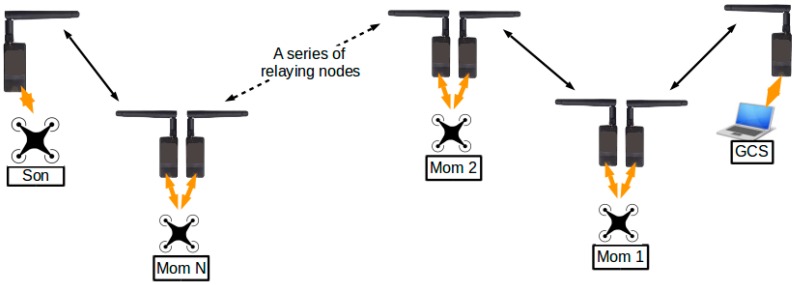
Extending the relay system by adding relaying nodes.

The current work focused only on communication relay through a single relaying node (UAV “Mom”). Relays through multiple relaying nodes, although feasible, will be conducted in the near future. The current configuration of the system allows the system to be extended by adding more relaying nodes. A possible configuration is illustrated in Figure 23, in which multiple relaying nodes (“Mom 1” to “Mom N”) are connected in series, forming a bidirectional relay system. The current design of the data frame permits, at most, 13 relaying nodes between “Son” and the GCS. When the format of the data frames was designed, four binary bits were assigned to represent the “ID” of the UAV. With four bits to use, at most 16 unique IDs could be represented, among which UAV “Son”, GCS, and a special broadcasting ID occupied three places. Therefore, there were 13 unique IDs left, which could then be taken up by 13 multiple UAV “Moms”, theoretically. However, the maximum number of relaying nodes in practice may also be subjected to the constraint of the data rate. With more UAVs in the system, more information will be exchanged. If the data rate is kept unchanged, communication between UAVs and GCS might experience longer delays.

## Figures and Tables

**Table 1:** Telemetry Installation and Settings.

3DR Radio	Installed at	Frequency Hopping Setting
T1-1	GCS	hopping sequence 1
T1-2	UAV “Mom”	hopping sequence 1
T2-1	UAV “Mom”	hopping sequence 2
T2-2	UAV “Son”	hopping sequence 2

## Supplementary Materials

The following is available online at https://www.youtube.com/watch?v=HxN0oafNmzw, Video S1: Multiple UAVs Telecommunication Demonstration. https://github.com/YifanJiangPolyU/UAV-communication-relay, Source code 1: Mission control software source code. https://github.com/HKPolyU-UAV/Mini_GCS, Source code 2: Mini-GSC source code.

## Abbreviations

The following abbreviations are used in this manuscript:

BLOS	Beyond-Line-of-Sight
UAV	Unmanned Aerial Vehicle
GCS	Ground Control Station
RSSI	Received Signal Strength Indication
FHSS	Frequency-Hopping Spread Spectrum
TDM	Time Division Multiplex
UART	Universal Asynchronous Receiver and Transmitter
CRC	Cyclic Redundancy Checking
BER	Bit Error Rate
GUI	Graphic User Interface
